# Gallbladder Agenesis and False-Negative Functional Imaging: A Case Report

**DOI:** 10.7759/cureus.103413

**Published:** 2026-02-11

**Authors:** Sarwat N Farooqi

**Affiliations:** 1 General and Colorectal Surgery, New Cross Hospital, Wolverhampton, GBR

**Keywords:** diagnostic laparoscopy, endoscopic ultrasound (eus), gallbladder agenesis, hepatobiliary iminodiacetic acid, laparascopic cholecystectomy, magnetic resonance cholangiopancreatography (mrcp)

## Abstract

Gallbladder agenesis is a rare congenital anomaly that may present with biliary colic-like symptoms and is frequently misdiagnosed, as non-visualisation of the gallbladder on ultrasound is often attributed to contraction or chronic disease, and functional imaging may further obscure the diagnosis if anatomical absence is not first excluded. We report a case of a 46-year-old female with a 10-year history of recurrent right upper quadrant pain consistent with biliary colic, in whom multiple ultrasounds failed to visualise the gallbladder and showed no gallstones, with persistently normal liver function tests. Magnetic resonance cholangiopancreatography (MRCP) demonstrated complete gallbladder agenesis with an otherwise normal biliary tree; however, subsequent endoscopic ultrasound and hepatobiliary scintigraphy suggested the presence of a functioning gallbladder with a high ejection fraction. Based on persistent symptoms and apparently reassuring functional imaging, laparoscopic cholecystectomy was undertaken, during which a complete absence of the gallbladder and cystic duct was confirmed intraoperatively. This case demonstrates that functional nuclear medicine imaging may actively contradict definitive anatomical imaging, falsely implying a normally functioning gallbladder in patients with gallbladder agenesis due to unimpeded bile flow into the small intestine. MRCP was the only modality that correctly identified the diagnosis, and early consideration of gallbladder agenesis with prioritisation of MRCP in patients with biliary-type pain and repeated gallbladder non-visualisation on ultrasound may prevent unnecessary surgery.

## Introduction

Gallbladder agenesis (GA) is a congenital structural abnormality characterised by the absence of a gallbladder. This can often present alongside similar structural abnormalities, such as imperforate anus and congenital heart lesions, with the incidence of GA thought to be <0.1% [1,2]. While imaging such as ultrasound and, more recently, magnetic resonance cholangiopancreatography (MRCP) scans can be useful in identifying this abnormality, it has often been traditionally diagnosed incidentally at laparotomy or laparoscopy. Ultrasound remains the first-line modality but often fails to visualise an absent gallbladder, which can be misinterpreted as a contracted or fibrotic gallbladder. Functional nuclear medicine studies are rarely described in the diagnostic pathway. This case highlights how functional results can generate false reassurance or misdirection even if anatomical absence is excluded beforehand.

## Case presentation

A 46-year-old female patient presented multiple times to the emergency department with symptoms of biliary colic, including right upper quadrant pain radiating to the back, with nausea/vomiting and loose stools. The presentations were often accompanied by an abdominal ultrasound report unable to visualise the gallbladder or presuming the gallbladder to be atrophic, with normal liver function tests. These presentations were on a background of a 10-year history of episodic right upper quadrant pain. Her symptoms were highly suggestive of a biliary pathology, which is often how she was treated, despite repeated ultrasounds being unable to identify the gallbladder. She had no jaundice, fever, or alcohol excess. Examination between episodes was unremarkable (Table 1).

**Table 1:** Serial biochemical laboratory results from consecutive presentations.

Biochemistry	06/11/2024 – 2 weeks prior to laparoscopic cholecystectomy	14/10/25 – Previous admission	Reference ranges
Sodium	136	137	133-146 mmol/L
Potassium	4.2	4.2	3.5-5.3 mmol/L
Estimated glomerular filtration rate (eGFR)	>90	>90	>59 mL/min/1.73 m^2^
Alanine aminotransferase (ALT)	17	17	0-55 IU/L
Alkaline phosphatase (ALP)	62	51	30-130 IU/L
Bilirubin	10	17	5-26 umol/L
Amylase	57	-	28–118 U/L
C-reactive protein	1	2	0.0-5.0 mg/L
White cell count	8.50	7.8	4.0-11.0 x10^9^/L

Over the decade, she underwent multiple abdominal ultrasounds, none of which visualised gallstones or sludge. Reports consistently described either a “contracted gallbladder” or “gallbladder not well visualised,” without further clarification.

**Figure 1:**
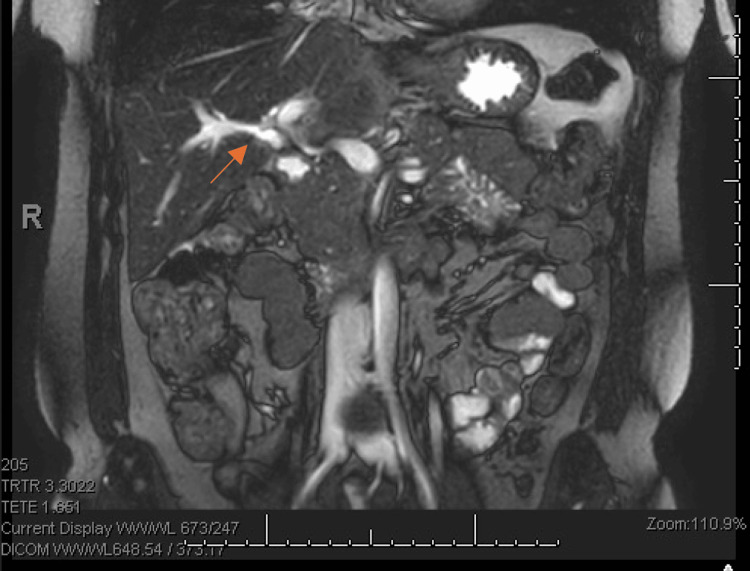
Coronal view of MRCP image showing agenesis of the gallbladder. Coronal view of magnetic resonance cholangiopancreatography (MRCP) demonstrating a normal-calibre common bile duct in the absence of gallbladder tissue, as shown by the arrow.

**Figure 2:**
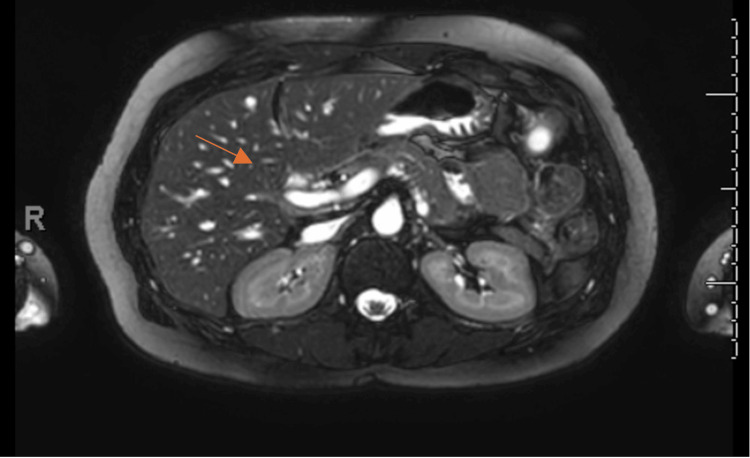
Sagittal view of MRCP showing a cross-section at the liver. Sagittal magnetic resonance cholangiopancreatography (MRCP) image demonstrating the absence of the gallbladder and cystic duct, as shown by the arrow.

She had been seen by both gastroenterology and general surgery regarding these symptoms and had been admitted under several upper GI consultants. Gastroenterology had been simultaneously investigating a possible diagnosis of colitis, which was confirmed via colonoscopy. Due to persistent symptoms and the absence of a radiological explanation, she was referred to hepatobiliary services. MRCP demonstrated complete agenesis of the gallbladder with proximal intrahepatic ducts appearing mildly prominent. The common bile duct (CBD) was tortuous and measured 3 mm in maximal calibre with no retained ductal stones. The common hepatic duct (CHD) measured 7 mm in calibre. These findings are shown in Figures 1, 2.

She was subsequently referred by gastroenterology for endoscopic ultrasound (EUS) based on her recurrent symptoms and likely GA on MRCP. This showed the following results: "gallbladder is thick-walled and contracted with sludge seen in it. Appearances are either chronic acalculous cholecystitis or adenomyomatosis". The report concluded that chronic inflammation is the cause of the imaging and recommended prompt referral to hepatobiliary surgeons for consideration of laparoscopic cholecystectomy.

In an attempt to correlate symptoms, a nuclear medicine hepatobiliary function scan (hepatobiliary iminodiacetic acid, HIDA) and stimulation scan were performed by general surgeons. The report described the gallbladder ejection fraction as 96% with prompt initial gallbladder isotope uptake. The scan found that the presumed gallbladder "empties satisfactorily, with dysfunction unlikely". The scan also found the "isotope entering the biliary tree empties promptly into proximal small bowel; ampullary hold-up unlikely". This gave the impression that the gallbladder was, in fact, present, albeit atrophic and was completely functional. This, alongside endoscopy of chronic acalculous cholecystitis, gave reasonable evidence to suggest laparoscopic cholecystectomy as the definitive management of the patient’s recurrent attacks.

**Figure 3:**
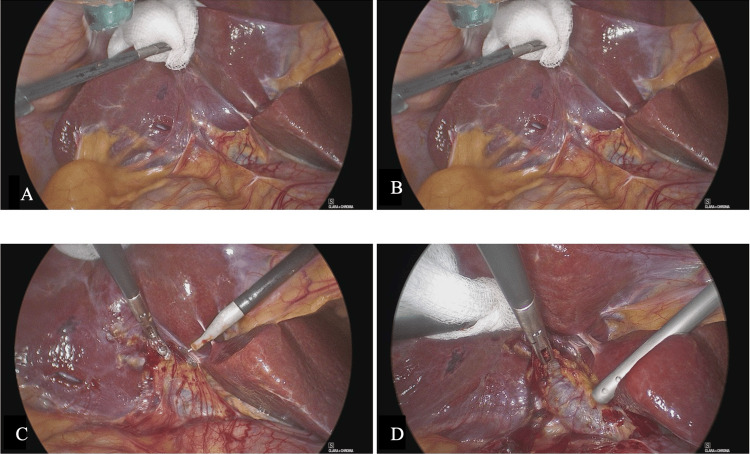
Intra-operative pictures confirming the diagnosis of gallbladder agenesis. Panels C and D show a tortuous common bile duct. (A) Absence of the gallbladder and cystic duct within the gallbladder fossa. (B) Additional operative view demonstrating normal surrounding anatomy without biliary variants. (C) Close intra-operative view of the hepatobiliary region confirming absence of gallbladder tissue. (D) Tortuous common bile duct visualised intra-operatively in the absence of a cystic artery.

The option was presented to the patient in the clinic by the lead surgeon, as EUS had confirmed the presence of a gallbladder, as well as satisfactory emptying seen on HIDA scanning. Given the ongoing biliary-type pain and failure of medical therapy, the patient elected to proceed with a laparoscopic cholecystectomy. Diagnostic laparoscopy was performed with the intent to remove the gallbladder. Intraoperatively, the surgeon confirmed the complete absence of the gallbladder, cystic duct, and cystic artery. Adhesiolysis was unnecessary, and no biliary anatomical variants were identified. No further surgical intervention was required. The findings were explained to the patient postoperatively, and she was subsequently referred back to gastroenterology for further investigation. Intra-operative images of the liver bed are shown in Figure 3.

## Discussion

Gallbladder agenesis is rare and often misdiagnosed. Most patients with symptoms present similarly to those with cholelithiasis, with research suggesting this may likely be due to biliary dyskinesia or sphincter of Oddi dysfunction [3,4].

Ultrasound remains the first-line imaging modality for suspected gallbladder pathology; however, its limitations in diagnosing gallbladder agenesis are well documented. It can often be misleading, as a non-visualised gallbladder may be incorrectly attributed to contraction or technical limitations [5]. This case shows evidence to consider gallbladder agenesis or an alternative diagnosis early when faced with a repeatedly non-visualised gallbladder on ultrasound. This case reinforces that repeated non-visualisation of the gallbladder, particularly in patients with normal liver biochemistry, should be considered a diagnostic red flag rather than a benign finding, prompting further anatomical evaluation rather than repeated ultrasound assessment.

Another point of discussion would be the most appropriate imaging technique to use for this condition. This case finds MRCP to be the most sensitive, with it being the only scan this patient underwent that showed a diagnosis of gallbladder agenesis. MRCP is often found to be the best imaging modality, with ultrasound, endoscopic retrograde cholangiopancreatography (ERCP), and HIDA scans repeatedly yielding ambiguous or false results, such as an atrophic gallbladder or high gallbladder ejection fraction in the absence of a gallbladder [6,7]. This case supports the earlier use of MRCP in patients with persistent biliary-type symptoms and repeated gallbladder non-visualisation, to avoid further invasive investigation and potential patient harm.

A particularly novel aspect of this case is the misleading role of functional imaging. HIDA scanning uses a radiotracer to follow the bilirubin metabolic pathway and excretion, making it a useful tool to assess gallbladder pathology [8]. HIDA scanning specifically has been reported to find inadequate uptake and no visualisation of the gallbladder in cases of gallbladder agenesis [9-11]. In this case, a HIDA scan was used after MRCP to confirm this diagnosis and falsely showed adequate isotope uptake and ejection. Based on these findings, a HIDA scan should not be used to rule out gallbladder agenesis, as findings can still be seemingly normal in the absence of a gallbladder.

The normal HIDA scan result is a seemingly rare finding, as most case reports of the same condition tend to show HIDA scanning correlating with the absence of a storage organ. The mechanism underlying this false-positive appearance likely reflects uninterrupted bile movement through the biliary tree in the absence of a storage organ. Tracer accumulation within the biliary ducts or proximal bowel may be misinterpreted as gallbladder activity, particularly when anatomical correlation is not rigorously applied. However, it is difficult to find an explanation for the prompt isotope uptake and satisfactory emptying of the gallbladder itself found in the report. One such study has found that false-negative HIDA results can be due to a loop of bowel mimicking the gallbladder or congenital abnormalities, which could be a possible explanation for this patient’s result [12]. This could also correlate with interpretation error or functional difficulties performing the scan. This case, therefore, highlights that HIDA scans should not be used to rule out gallbladder agenesis and that functional imaging must always be interpreted in conjunction with definitive anatomical studies.

Furthermore, whilst most cases of GA are discovered intra-operatively, this case highlights that adequate pre-operative imaging can negate the need for surgery. Many cases of GA have shown patients undergoing surgical procedures due to a clinical diagnosis based on symptoms and ambiguous ultrasound imaging. However, this case had evidence of GA in the form of MRCP, which was confounded by EUS and HIDA scanning results. As almost all cases of gallbladder agenesis have shown false-positive HIDA results, this gave a strong reason to proceed with cholecystectomy in this case. The decision to proceed with surgery reflected the complexity of the diagnostic pathway rather than inappropriate clinical management, and this case is presented to highlight recognised imaging limitations and suggests that when anatomical and functional imaging conflict, anatomical imaging should take diagnostic precedence to avoid unnecessary intervention.

This case further illustrates that gallbladder agenesis should be considered not only as a structural abnormality but also as a mimic of functional biliary disorders, explaining persistent symptoms despite normal laboratory results and absence of gallstones. Recognising this distinction may allow earlier transition to conservative management strategies, streamlining to the correct speciality and reducing patient morbidity associated with repeated admissions and invasive procedures.

This case emphasises that when ultrasound repeatedly fails to visualise the gallbladder, agenesis should be considered early, and MRCP should be performed before nuclear medicine studies or surgical referral.

What makes this case different?

Gallbladder agenesis was correctly identified on MRCP prior to surgery, yet subsequent functional imaging falsely suggested a present and functioning gallbladder. Furthermore, hepatobiliary scintigraphy demonstrated a high gallbladder ejection fraction despite complete anatomical absence, a rarely reported diagnostic pitfall. This highlights how functional imaging can actively contradict anatomical imaging, leading to unnecessary surgery. This emphasises the importance of prioritising anatomical imaging when investigations are discordant.

Ethical approval

Ethical approval was not required for this case report in accordance with institutional policy, as it describes a single patient without experimental intervention. Written informed consent was obtained from the patient for publication of this case and accompanying images.

## Conclusions

Gallbladder agenesis is a rare but important differential diagnosis in patients presenting with biliary-type pain and repeated non-visualisation of the gallbladder on ultrasound. This case demonstrates that functional imaging, including hepatobiliary scintigraphy, may be misleading and can falsely imply the presence of a normally functioning gallbladder in the absence of the organ. MRCP was the only investigation that accurately identified gallbladder agenesis in this patient. Early consideration of this diagnosis, prioritisation of anatomical imaging over functional studies when results are discordant, and careful multidisciplinary interpretation may prevent unnecessary surgical intervention and reduce patient morbidity.
